# Clopidogrel response predicts thromboembolic events associated with coil embolization of unruptured intracranial aneurysms: A prospective cohort study

**DOI:** 10.1371/journal.pone.0249766

**Published:** 2021-04-08

**Authors:** Eiji Higashi, Shoji Matsumoto, Ichiro Nakahara, Taketo Hatano, Akira Ishii, Nobutake Sadamasa, Tsuyoshi Ohta, Takuma Ishihara, Keisuke Tokunaga, Mitsushige Ando, Hideo Chihara, Konosuke Furuta, Tetsuya Hashimoto, Koji Tanaka, Kazutaka Sonoda, Junpei Koge, Wataru Takita, Takuro Hashikawa, Yusuke Funakoshi, Daisuke Kondo, Takahiko Kamata, Atsushi Tsujimoto, Takuya Matsushita, Hiroyuki Murai, Keitaro Matsuo, Takanari Kitazono, Junichi Kira

**Affiliations:** 1 Department of Neurosurgery, Stroke Center, Kokura Memorial Hospital, Kitakyushu, Japan; 2 Department of Neurology, Stroke Center, Kokura Memorial Hospital, Kitakyushu, Japan; 3 Department of Neurosurgery, Kyoto University Graduate School of Medicine, Kyoto, Japan; 4 Department of Neurosurgery, Stroke Center, Koseikai Takeda Hospital, Kyoto, Japan; 5 Department of Neurosurgery, National Cerebral and Cardiovascular Center, Osaka, Japan; 6 Innovative and Clinical Research Promotion Center, Gifu University Hospital, Gifu, Japan; 7 Department of Cerebrovascular Medicine and Neurology, National Hospital Organization Kyushu Medical Center, Fukuoka, Japan; 8 Department of Neurosurgery, Shiga General Hospital, Moriyama, Japan; 9 Department of Neurology, University of California, Los Angeles, Los Angeles, CA, United States of America; 10 Department of Neurology, Graduate School of Medical Sciences, Kyushu University, Fukuoka, Japan; 11 Department of Neurology, Fukuoka Saiseikai General Hospital, Fukuoka, Japan; 12 Department of Cerebrovascular Medicine, National Cerebral and Cardiovascular Center, Osaka, Japan; 13 Department of Neurology, National Hospital Organization Nagoya Medical Center, Nagoya, Japan; 14 Department of Neurosurgery, St. Mary’s Hospital, Kurume, Japan; 15 Department of Neurosurgery, Graduate School of Medical Sciences, Kyushu University, Fukuoka, Japan; 16 Department of Neurology, Japan Community Health Care Organization Kyushu Hospital, Kitakyushu, Japan; 17 Department of Neurology, International University of Health and Welfare, Narita, Japan; 18 Division of Cancer Epidemiology and Prevention, Aichi Cancer Center Research Institute, Nagoya, Aichi, Japan; 19 Department of Cancer Epidemiology, Nagoya University Graduate School of Medicine, Nagoya, Aichi, Japan; 20 Department of Medicine and Clinical Science, Graduate School of Medical Sciences, Kyushu University, Fukuoka, Japan; Osaka University Graduate School of Medicine, JAPAN

## Abstract

**Objective:**

Periprocedural thromboembolic events are a serious complication associated with coil embolization of unruptured intracranial aneurysms. However, no established clinical rule for predicting thromboembolic events exists. This study aimed to clarify the significance of adding preoperative clopidogrel response value to clinical factors when predicting the occurrence of thromboembolic events during/after coil embolization and to develop a nomogram for thromboembolic event prediction.

**Methods:**

In this prospective, single-center, cohort study, we included 345 patients undergoing elective coil embolization for unruptured intracranial aneurysm. Thromboembolic event was defined as the occurrence of intra-procedural thrombus formation and postprocedural symptomatic cerebral infarction within 7 days. We evaluated preoperative clopidogrel response and patients’ clinical information. We developed a patient-clinical-information model for thromboembolic event using multivariate analysis and compared its efficiency with that of patient-clinical-information plus preoperative clopidogrel response model. The predictive performances of the two models were assessed using area under the receiver-operating characteristic curve (AUC-ROC) with bootstrap method and compared using net reclassification improvement (NRI) and integrated discrimination improvement (IDI).

**Results:**

Twenty-eight patients experienced thromboembolic events. The clinical model included age, aneurysm location, aneurysm dome and neck size, and treatment technique. AUC-ROC for the clinical model improved from 0.707 to 0.779 after adding the clopidogrel response value. Significant intergroup differences were noted in NRI (0.617, 95% CI: 0.247–0.987, p < .001) and IDI (0.068, 95% CI: 0.021–0.116, p = .005).

**Conclusions:**

Evaluation of preoperative clopidogrel response in addition to clinical variables improves the prediction accuracy of thromboembolic event occurrence during/after coil embolization of unruptured intracranial aneurysm.

## Introduction

With the development of devices and techniques that facilitate endovascular treatment in recent years, the outcomes and therapeutic indications for unruptured intracranial aneurysms (UIA) have improved. Although periprocedural thromboembolic event (TE) is still a severe complication that occurs in 3.7–7.5% patients during/after coil embolization of UIA [1–4] and can lead to permanent neurological deficits [5], there is no established clinical rule for the prediction of TE. Thus, the prediction of TE remains challenging.

Dual antiplatelet therapy (DAT), most commonly with aspirin and clopidogrel, is recommended as the standard therapy for the prevention of TE in patients undergoing neurointervention [6, 7]. Response to clopidogrel varies widely among individuals, and low response to clopidogrel might be associated with periprocedural TE during coil embolization of UIA [1, 8–11]. However, no large-scale prospective studies have been performed to validate the cut-off value of clopidogrel response to predict TE; therefore, the importance of evaluation of preoperative clopidogrel response is still controversial [12].

Other than the clopidogrel response, previous studies have also shown that clinical variables such as an older age [2], posterior location [13], larger aneurysm [3, 14, 15], wide neck aneurysm [14], the use of adjunctive technique [14], and duration of procedure [16] are also associated with periprocedural TE with regard to coil embolization of UIA.

We hypothesized that it would be possible to increase the accuracy of TE prediction during/after coil embolization for UIA by adding the preoperative clopidogrel response value to clinical variables.

The purpose of this study was to clarify the significance of adding preoperative clopidogrel response value to the evaluation of clinical factors when predicting the occurrence of TE during/after coil embolization of UIA and to develop and validate a nomogram for the prediction of TE.

## Materials and methods

### Study design

This prospective, single-center, cohort study included patients undergoing elective coil embolization for UIA. Our institute is a core regional hospital for neuro-intervention and a high-volume cardiovascular disease center (2000 procedures per year). The sample size for this study was based on data availability.

### Patients

At our institute, we enrolled consecutive patients who were scheduled to undergo endovascular treatments for untreated UIA between July 2010 and October 2017. Exclusion criteria were missing platelet function test data, presence of a subarachnoid hemorrhage that required a craniotomy procedure because of intra-procedural rupture, and presence of large aneurysm treated with flow-diverter placement. The study was approved by the institutional review board of Kyushu University and Kokura Memorial Hospital and conducted in accordance with the ethical standards of the Declaration of Helsinki. Written informed consent was obtained from all patients included in the study or their families. The results of the study are reported in accordance with the Transparent Reporting of a multivariable prediction model for Individual Prognosis or Diagnosis (TRIPOD) statement.

### Definition of the periprocedural TE

The primary clinical outcome was the occurrence of TE, which was defined as intra-procedural thrombus formation and postprocedural symptomatic cerebral infarction with evidence obtained via postprocedural head magnetic resonance imaging (MRI) within 7 days after the procedure. In the intensive-care unit, a neurological examination was performed every 2 hours. If a new neurological deficit appeared, MRI of the head was performed to assess the appearance of a new cerebral infarct using a 1.5- or 3-Tesla MRI system.

### Data collection and definitions

Data on the clinical characteristics of participants, including age, sex, hypertension, dyslipidemia, diabetes mellitus, smoking, ischemic stroke history, aneurysm dome size, aneurysm neck size, location of aneurysms, and treatment technique, were obtained from our single-center prospective database. Hypertension was diagnosed based on a systolic blood pressure of ≥140 mmHg or a diastolic blood pressure of ≥90 mmHg in the chronic stage or from previous medical documents [17]. Dyslipidemia was diagnosed based on a low-density lipoprotein cholesterol level of ≥140 mg/dL, a high-density lipoprotein cholesterol level of <40 mg/dL, or a triglyceride level of ≥150 mg/dL or from previous medical documents [18]. Diabetes mellitus was diagnosed according to the diagnostic criteria of the Japan Diabetes Society or from previous medical documents [19]. Medical therapy was aggressively prescribed for all patients who met the clinical definitions of hypertension, dyslipidemia, or diabetes mellitus preoperatively. Smoking was defined according to either the current smoking status or a smoking history. Aneurysm location was defined at the origin of the neck on the basis of the classification of Bouthillier et al. [20]. Location of the aneurysm was classified into one of the following three categories: internal carotid artery, anterior or middle cerebral artery, and posterior circulation (posterior cerebral artery, basilar artery, or vertebral artery). Aneurysm dimensions were measured by 3D reformatted images derived from rotational catheter angiograms by using the Allura 3D-RA workstation (Philips Healthcare, Best, the Netherlands).

### Platelet function measurements

We evaluated preprocedural responses to clopidogrel and aspirin using the VerifyNow P2Y_12_ assay (Accumetrics, San Diego, CA, USA) [21]. Patients’ blood was collected from the median cubital vein using a 21-gauge blood collection needle within 24 hours before the procedure. The result output was expressed as P2Y12 reaction units (PRU).

### Coil embolization procedure

To prevent periprocedural TE, DAT (75 mg of clopidogrel and 100 mg of aspirin per day) was started at least 7 days before coil embolization. A loading dose of 300 mg clopidogrel was used for patients receiving antiplatelet drugs for less than 7 days. If the patients had already been taking aspirin or clopidogrel for their primary disease, then they were asked to continue the drugs, and if they were receiving aspirin, clopidogrel was added, and if they were receiving clopidogrel, aspirin was added. If the patient had already been receiving other antiplatelet drugs, then the drugs were preoperatively changed to aspirin and clopidogrel.

Surgical procedures were performed using biplane angiographic systems under general anesthesia. After inserting the sheath, the activated clotting time was checked hourly and maintained between 250 and 300 seconds by intravenously injecting heparin during the procedure. The decision to use stent-assist coiling was based on aneurysm morphology and aneurysm-parent vessel relationship. Treatment technique was classified into one of the following three categories: simple coiling, balloon-assisted coiling, and stent-assisted coiling (Neuroform [Stryker, Kalamazoo, MI, USA], Enterprise [Johnson & Johnson Codman, Miami, FL, USA], or low-profile visualized intraluminal support [MicroVention Terumo, Tustin, CA, USA]) After completing the procedure, patients were transferred to the intensive-care unit without reversal of heparinization. DAT was continued for at least 1 month post procedure in all the cases.

### Statistical analysis

Continuous variables are presented as median (interquartile range [IQR]), and categorical variables as frequency and percentage. We developed and compared two models to assess the performance of PRU in predicting TE. Variables such as patient clinical information which were previously reported to be associated with periprocedural TE, including age [2], aneurysm location [13], aneurysm dome size [3, 14, 15], aneurysm neck size [14], and treatment technique [14] were included in multivariable logistic regression to create the clinical model to predict TE, considering their clinical importance. The duration of the procedure [16] had also been reported to be associated with periprocedural TE. However, because this study aimed to identify predictors of TE that could be assessed before treatment, we did not include procedural duration in this analysis as it is a measure of treatment outcome. The clinical + PRU model was developed by adding the information of the preoperative PRU value (as a continuous variable) to the clinical model.

The prediction model was developed using data only from patients in whom all of the study variables had been assessed (n = 345). The discrimination performance of each model was assessed using the area under the receiver-operating characteristic curve (AUC-ROC). Bootstrap validation was performed with 150 resamples to validate and calibrate each prediction model. The bootstrap bias-corrected AUC (bootstrap AUC-ROC) was reported as the measure of the predictive performance of the model. The optimism [22] of each model was estimated using 150 bootstrap resamples. Optimism assesses the magnitude of overfitting of logistic regression model (a value less than 0.3 is considered as good) and was calculated using C-statistics by bootstrap samples. Using net reclassification improvement (NRI) and integrated discrimination improvement (IDI), we assessed whether there was a difference in the diagnostic ability between the two models. The total NRI was the summation of the accurate reclassifications of patients with and without TE. In the patients with TE, improvement of reclassification was the difference between the percentage of patients reclassified as a higher risk group and that of patients reclassified as a lower risk group. Similarly, in the patients without TE, improvement of reclassification was the difference between the percentage of patients reclassified as a lower risk group and that of patients reclassified as a higher risk group. The total IDI provides the difference in mean predicted probabilities, representing the amount by which addition of a variable to a model increases the separation of the mean predicted probabilities for TE and non-TE [23]. To confirm the threshold for determining TE, we calculated the cut-off value of PRU using an ROC curve. Predicted probability of TE was provided by a multivariable logistic regression model when covariates including age, sex, hypertension, diabetes mellitus, dyslipidemia, smoking, aneurysm dome size, neck size, and aneurysm location were fixed at the median. To make the final model easier to use in a clinical setting, we developed a nomogram. The goodness-of-fit of the model was confirmed using the AIC and Hosmer-Lemeshow test. The average causal mediation effects regarding procedure duration were evaluated. The statistical analyses were performed using R version 3.6.2 (R Foundation for Statistical Computing, Vienna, Austria). A two-sided p value < .05 was considered statistically significant.

## Results

**Fig 1:**
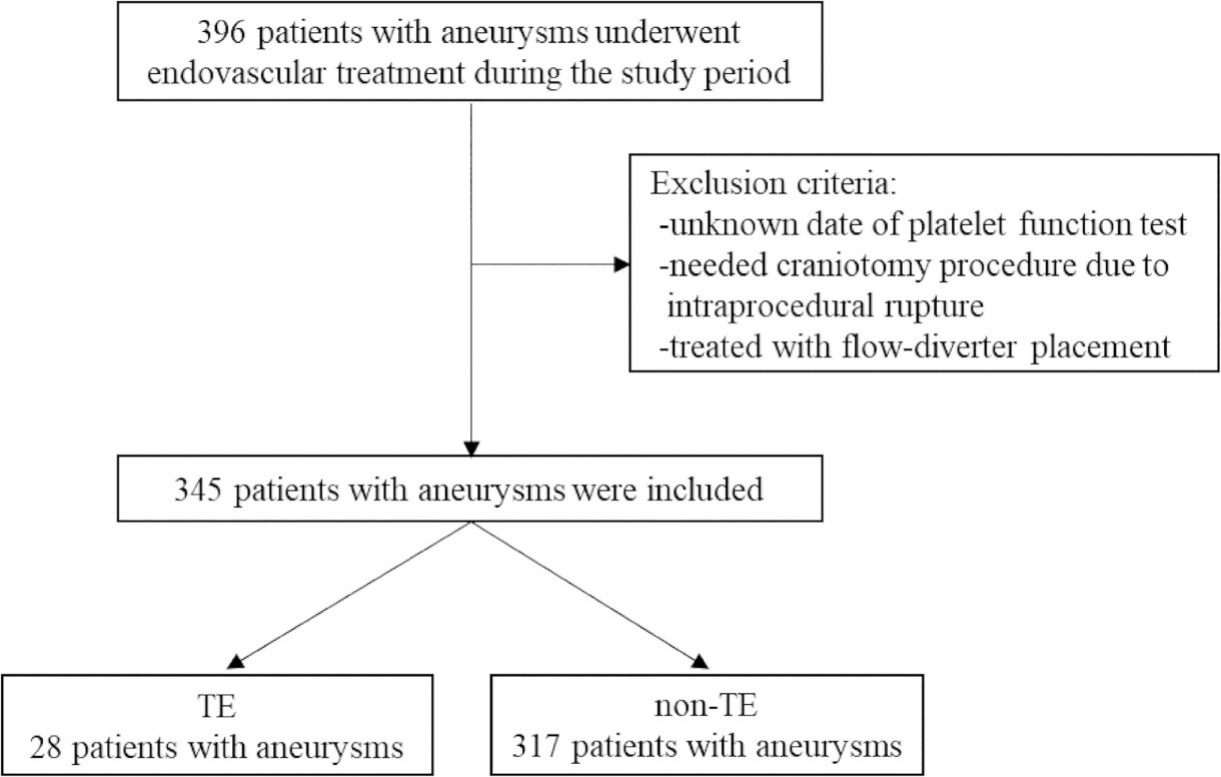
Flowchart showing patient participation. Abbreviations: TE, thromboembolic event.

There were 28 patients in the TE group and 317 in the non-TE group (Fig 1). The baseline characteristics of the patients in the two groups are shown in Table 1.

**Table 1:** Baseline characteristics of patients in the TE and non-TE groups.

	Total (n = 345)	TE group (n = 28)	non-TE group (n = 317)	P Value
Age, median [IQR], years	64 (54, 72)	66 (60, 75)	63 (53, 72)	0.055
Female, n (%)	270 (78.3%)	23 (82.1%)	247 (77.9%)	0.603
Hypertension, n (%)	203 (58.8%)	23 (82.1%)	180 (56.8%)	0.009
Dyslipidemia, n (%)	117 (33.9%)	10 (35.7%)	107 (33.8%)	0.834
Diabetes mellitus, n (%)	35 (10.1%)	6 (21.4%)	29 (9.1%)	0.039
Smoking, n (%)	113 (32.8%)	11 (39.3%)	102 (32.2%)	0.442
Ischemic stroke history, n (%)	15 (4.3%)	2 (7.1%)	13 (4.1%)	0.449
Preprocedural PRU, median [IQR]	223 (162, 274)	286 (230, 327)	219 (158, 265)	0.001
Dome size, median [IQR], mm	5.9 (4.9, 7.5)	6.9 (5.4, 10.3)	5.9 (4.8, 7.5)	0.061
Neck size, median [IQR], mm	3.8 (3.0, 4.8)	4.5 (3.1, 5.7)	3.7 (3.0, 4.7)	0.120
Aneurysm location				0.064
ICA, n (%)	213 (61.7%)	12 (42.9%)	201 (63.4%)	
ACA/MCA, n (%)	70 (20.3%)	7 (25.0%)	63 (19.9%)	
Posterior circulation, n (%)	62 (18.0%)	9 (32.1%)	53 (16.7%)	
Treatment technique				0.345
Simple coiling, n (%)	80 (23.2%)	5 (17.9%)	75 (23.7%)	
Balloon-assisted coiling, n (%)	101 (29.3%)	6 (21.4%)	95 (30.0%)	
Stent-assisted coiling, n (%)	164 (47.5%)	17 (60.7%)	147 (46.4%)	
Duration of procedure, median [IQR], min	134 (105, 180)	155 (123, 243)	131 (102, 175)	0.001

The data are presented as the number (percentage) or median (interquartile range).

Abbreviations: TE, thromboembolic event; PRU, P2Y_12_ reaction units; IQR, interquartile range; ICA, internal carotid artery; ACA, anterior cerebral artery; MCA, middle cerebral artery

**Fig 2:**
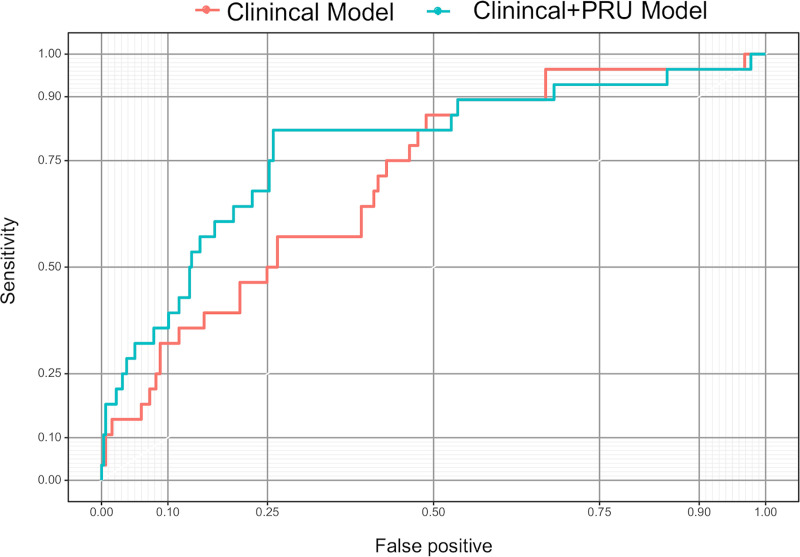
Receiver-operating characteristic curves for the clinical model and clinical + PRU model. Abbreviations: PRU, P2Y_12_ reaction units.

**Fig 3:**
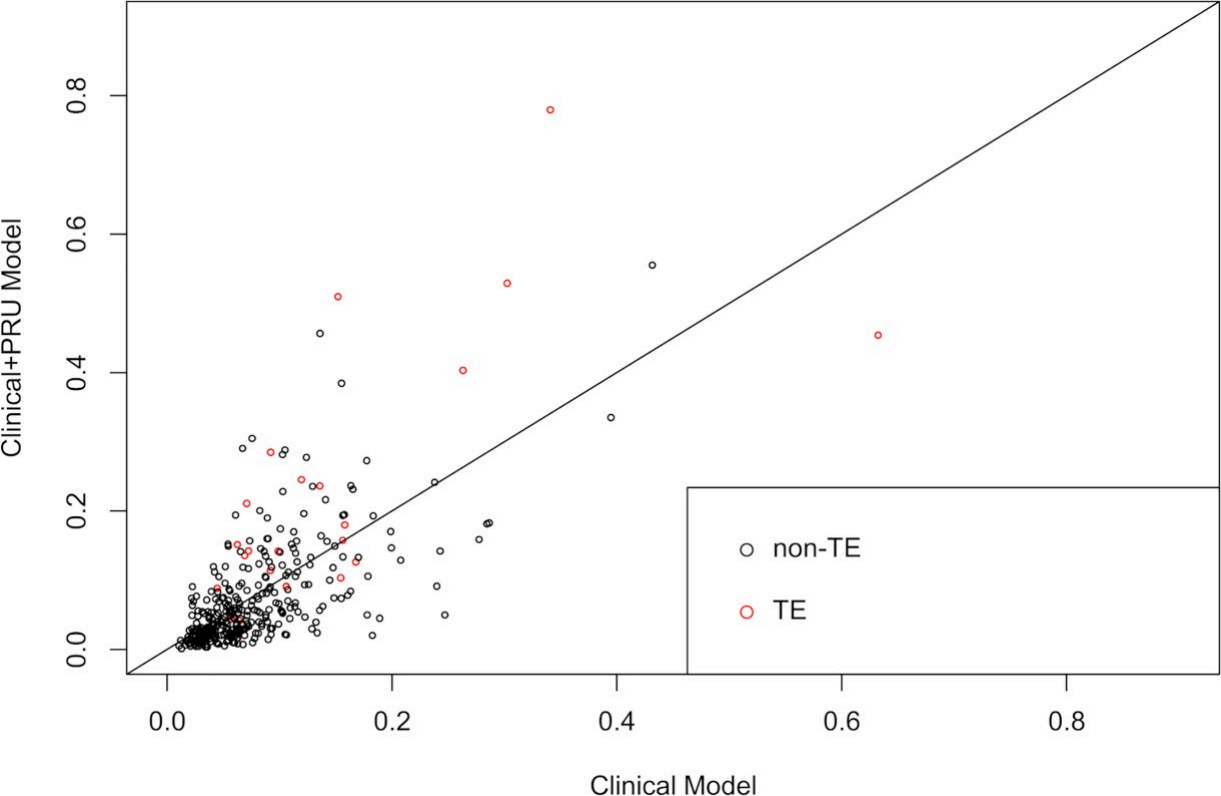
Comparison between the clinical model and clinical+ PRU model using the net reclassification index and integrated discrimination index. Scatter plot showing the predicted probability of infarction as shown by the Clinical model and Clinical+ PRU model. The red and black circles indicate patients with and without TE, respectively. Abbreviations: TE, thromboembolic event; PRU, P2Y_12_ reaction units.

The clinical prediction rule was developed using two multivariable logistic regression models: a clinical model, which included age, aneurysm location, aneurysm dome size, aneurysm neck size, and treatment technique; and a clinical + PRU model that included age, aneurysm location, aneurysm dome size, aneurysm neck size, treatment technique, and preoperative PRU value. The AICs of the clinical model and the clinical + PRU model were 194.7 and 182.3, respectively, and the p-values of the Hosmer-Lemeshow test were 0.1 and 0.589, respectively. The ROC curves for the clinical model and clinical + PRU model are shown in Fig 2. The model-based predicted probability of TE fitted well with the observed data, and discrimination ability, which was assessed through bootstrap with an area under the ROC curve, improved from 0.707 [95% confidence interval (95% CI): 0.612–0.802) to 0.779 (95% CI: 0.679–0.878] after adding the PRU value. Bootstrap AUC-ROC values of the clinical and clinical + PRU models were 0.656 and 0.744, respectively. The corresponding estimated optimism values were 0.351 and 0.269, respectively, indicating that there was no evidence of obvious overfitting in the clinical + PRU model. We assessed the additive predictive ability of PRU value by comparing the diagnostic abilities of the clinical and clinical + PRU models using NRI and IDI, and both these values were statistically significant (NRI: 0.617, 95% CI: 0.247–0.987, P < .001; IDI: 0.0682, 95% CI: 0.0208–0.116, P = .005) (Fig 3). We found that the clinical + PRU model appeared to be superior to the clinical model.

**Fig 4:**
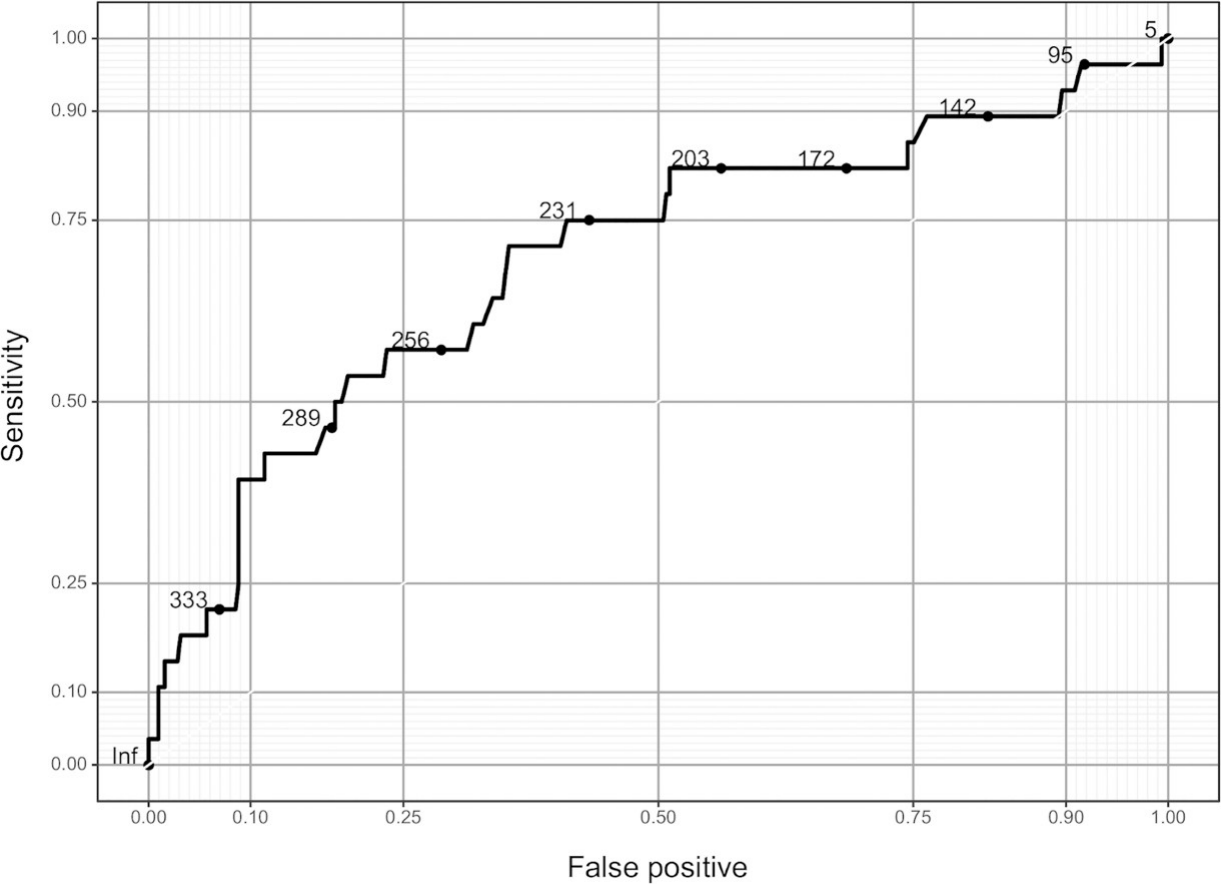
Area under the receiver-operating characteristic curve for the PRU only model. Abbreviations: PRU, P2Y_12_ reaction units.

**Fig 5:**
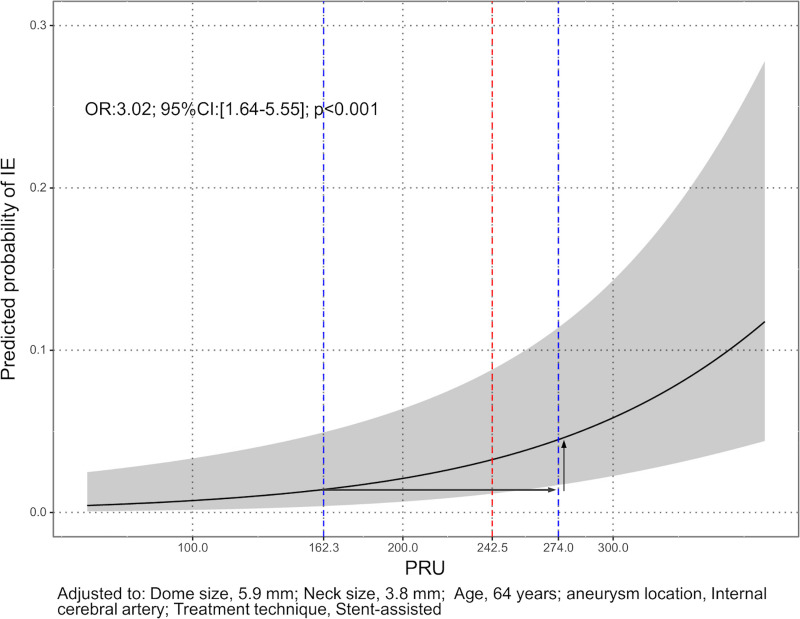
Relationship between the predicted probability of TE and PRU value adjusted for values of the clinical model. Predicted probability of infarction was provided by a multivariable logistic regression model when covariates including age, sex, hypertension, diabetes mellitus, dyslipidemia, smoking, aneurysm dome size, neck size, and aneurysm location were fixed at the median. The gray area indicates the 95% CI of predicted probability by PRU. The arrows indicate, for example, that if there is an increase in PRU from the 25^th^ percentile (162) to the 75^th^ percentile (274), the odds of TE increase by approximately 3-fold (Odds ratio: 3.02; 95% CI: [1.64–5.55]; P < .001). Abbreviations: TE, thromboembolic event; CI, confidence interval; PRU, P2Y_12_ reaction units.

The ROC curve for only the preoperative PRU value is shown Fig 4. The sensitivity was 0.714 and specificity was 0.647 for a threshold PRU value of 243. The relationship between the predicted probability of TE and PRU value after adjusting for values of the clinical model is shown in Fig 5. The arrows indicate, for example, that if there is an increase in PRU from the 25th percentile (162) to the 75th percentile (274), the odds of TE increase by approximately 3-fold (Odds ratio: 3.02; 95% CI: [1.64–5.55]; P < .001).

**Fig 6:**
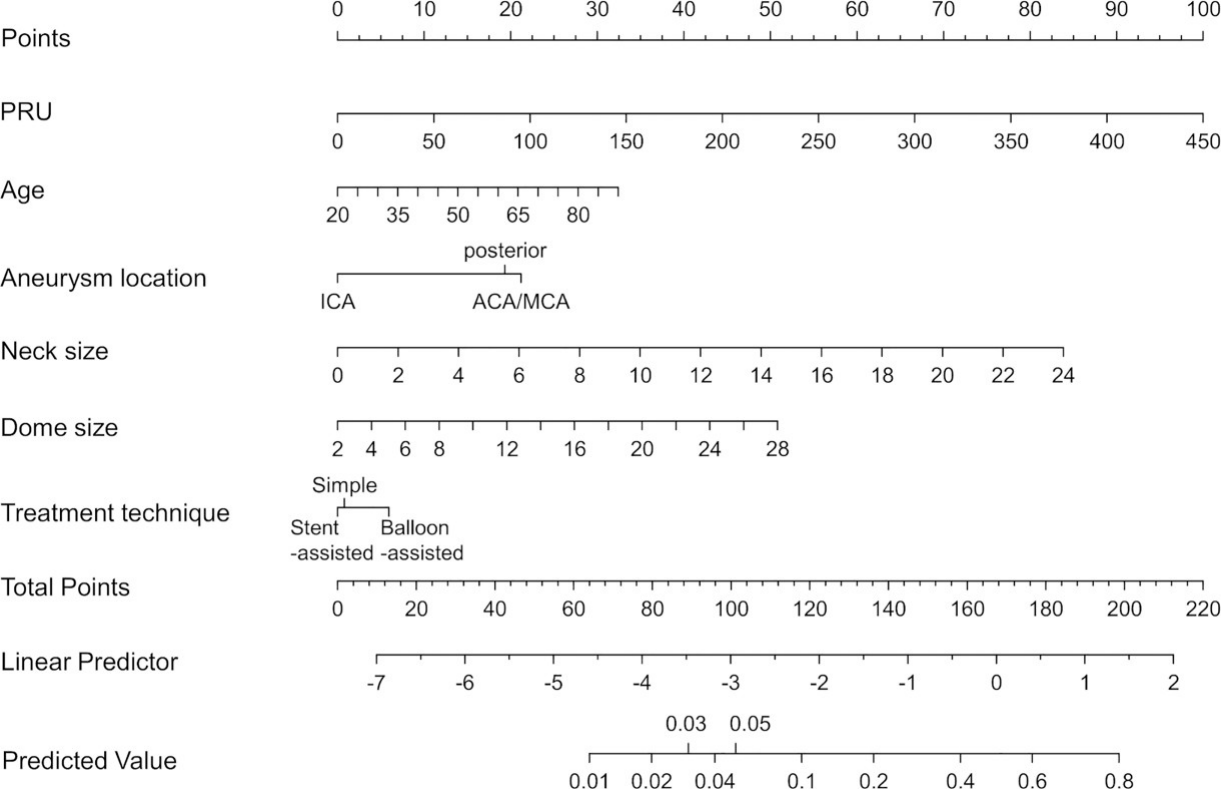
Nomogram that predicts the occurrence of TE during/after coil embolization of UIA using the clinical + PRU model. Abbreviations: TE, thromboembolic event; PRU, P2Y_12_ reaction units; UIA, unruptured intracranial aneurysms.

Finally, we created a nomogram to predict the occurrence of TE during/after coil embolization of UIA using the clinical + PRU model with an improved ease of use in the clinical setting (Fig 6). Use of the nomogram is simple. For example, if the PRU value is 270 (60 points), aneurysm dome size is 8 mm (11 points), aneurysm neck size is 4 mm (14 points), ICA location (0 points), a balloon-assisted technique is used (5 points), and the patient is 42‐years‐old (10 points), a total point value of 100 is given, which corresponds to a 5% risk of perioperative TE during/after coil embolization of UIA.

The results of logistic regression analysis for all models and the clinical + PRU + procedure duration model are shown in S1 Table. Causal mediating analysis did not confirm the average causal mediation effects of procedure duration (p = 0.6).

## Discussion

In this study, we have shown that the evaluation of preoperative clopidogrel response in additional to clinical variables improves the accuracy of predicting the occurrence of TE during/after coil embolization of UIA. Furthermore, we have developed a preoperative nomogram that requires patient clinical information and clopidogrel response to predict the occurrence of TE during/after coil embolization of UIA. To our knowledge, this is the first large-scale, prospective study to evaluate clopidogrel response with future TE in patients who underwent coil embolization of UIA.

Previous studies have shown that periprocedural symptomatic TE occurred in 3.7–7.5% patients during/after coil embolization of UIA [1–4]. In this study, the percentage of patients who developed TE was 8.1%, which was largely consistent with previous reports.

In this study, we used the VerifyNow P2Y12 assay to assess preprocedural clopidogrel response. This assay correlates strongly with light transmittance aggregometry; it is the gold standard for the quantification of platelet reactivity in patients treated with clopidogrel, prasugrel, or ticagrelor [21, 24, 25]. The VerifyNow P2Y12 assay is widely used in large studies in the field of percutaneous coronary artery intervention [26–29]; however, it has not proven to be useful in neurointervention approaches [12].

As for the cut-off value of PRU for predicting the occurrence of TE during/after coil embolization for UIA, two studies showed cut-off values of 220 PRU [11] and 295 PRU [1], and in this study, we found that the threshold PRU value was 243 and that an increase in PRU by one IQR was associated with an approximately 3-fold increase in odds of TE after adjusting for clinical factors. The differences in the clinical conditions of each study may have contributed to the wide variation in cut-off values.

In this study, we found that preprocedural clinical + PRU model was superior to the clinical risk factor model in predicting TE after coil embolization of UIA. In other words, the risk of TE increases with an increase in the PRU value in addition to that in the clinical factors. The current study found that combining clinical findings with information on clopidogrel reactivity could help predict TE more accurately.

The nomogram developed in this study showed that several clinical factors other than clopidogrel response were associated with perioperative TE during/after coil embolization for UIA. Older age [2], aneurysm location [13], larger aneurysm size [3, 14, 15], and larger aneurysm neck size [14], this finding is consistent with that of previous reports, and we have reaffirmed the importance of simultaneous consideration of clinical factors and clopidogrel response in predicting perioperative TE. On the other hand, among the factors reported to be associated with TE, adjunctive technique is more weakly associated than PRU.

In this study, we have demonstrated the importance of evaluating clopidogrel responsiveness using VerifyNow before surgery. Furthermore, we have developed and validated nomograms using clopidogrel responses for the prediction of TE during/after coil embolization of UIA. Our nomogram can calculate the approximate probability of the occurrence of TE. Previous reports, including the results of our study, have shown an incidence of TE of ~ 8% or less. If the nomogram shows a rate of 8% or more, more stringent adjustment of the PRU may be necessary. Since PRUs are the most relevant in predicting the risk of developing TE and can be modified by adding cilostazol [30] or by replacing clopidogrel with prasugrel [31], it is desirable to adjust them at least to a cut-off value. We believe that this nomogram could facilitate safer coil embolization of UIA.

The present study has several limitations. First, the present results might be specific to our techniques and equipment because the current study employed a single-center design. Second, preprocedural PRUs were not successfully calculated in all patients. Third, the present study is at risk of being statistically underpowered because of the relatively small number of patients with TE. The prospective validation of the cut-off PRU value for predicting TE is also required. Fourth, the type of coil was not considered in the present study; the use of different types of coils may affect the occurrence of periprocedural thromboembolism, as described previously [32]. Further prospective multicenter studies are needed to confirm these data and to evaluate whether clinical + PRU model is the best approach for predicting TE after coil embolization of UIA.

## Conclusions

We have showed that evaluation of preoperative clopidogrel response in addition to clinical variables improves the accuracy of predicting TE occurrence during/after coil embolization of UIA. This information may permit more accurate risk stratification on an individual case basis before treatment.

## Supporting information

S1 Table Logistic regression analysis results for the clinical model, clinical + PRU model, and clinical + PRU + procedure duration model. (DOCX)

S2 Table Database containing patient information. (PDF)
