# Supplementary material for: Clopidogrel response predicts thromboembolic events associated with coil embolization of unruptured intracranial aneurysms: A prospective cohort study
Source: PLoS One. 2021 Apr 8;16(4):e0249766. doi: 10.1371/journal.pone.0249766 (PMC8031457; doi:10.1371/journal.pone.0249766)
Supplement: S1 Table — (DOCX) [file pone.0249766.s001.docx]

|  | Clinical Model | | Clinical + PRU Model | | Clinical + PRU + Procedure Duration Model | |
| --- | --- | --- | --- | --- | --- | --- |
| Variable | OR [95% CI] | P value | OR [95% CI] | P value | OR [95% CI] | P value |
| PRU | - | - | 1.01 [1, 1,02] | <0.001 | 1.01 [1.01, 1.02] | <0.001 |
| Procedure duration | - | - | - | - | 1.86 [1.23, 2.82] | 0.004 |
| Age | 1.03 [0.99, 1.07] | 0.149 | 1.02 [0.98, 1.06] | 0.302 | 1.03 [0.99, 1.07] | 0.184 |
| Neck size | 1.06 [0.88, 1.29] | 0.526 | 1.17 [0.95, 1.43] | 0.132 | 1.24 [0.99, 1.55] | 0.057 |
| Dome size | 1.1 [0.99, 1.24] | 0.087 | 1.09 [0.97, 1.22] | 0.135 | 0.99 [0.86, 1.13] | 0.851 |
| Aneurysm location posterior | 2.3 [0.81, 6.51] | 0.116 | 2.36 [0.79, 7.03] | 0.123 | 1.92 [0.62, 5.94] | 0.258 |
| Aneurysm location ACA/MCA | 2.21 [0.77, 6.36] | 0.141 | 2.56 [0.85, 7.77] | 0.096 | 2.05 [0.66, 6.37] | 0.213 |
| Treatment technique Simple | 0.94 [0.29, 3.11] | 0.923 | 1.04 [0.29, 3.66] | 0.957 | 1.19 [0.33, 4.26] | 0.784 |
| Treatment technique Balloon assisted | 1.13 [0.38, 3.37] | 0.833 | 1.3 [0.42, 4.07] | 0.651 | 1.23 [0.39, 3.85] | 0.723 |
