# Supplementary material for: Clopidogrel response predicts thromboembolic events associated with coil embolization of unruptured intracranial aneurysms: A prospective cohort study
Source: PLoS One. 2021 Apr 8;16(4):e0249766. doi: 10.1371/journal.pone.0249766 (PMC8031457; doi:10.1371/journal.pone.0249766)
Supplement: S2 Table — (PDF) [file pone.0249766.s002.pdf]

Age	sex	Aneurysms location	Dome size(mm)	Neck size(mm)	Treatment technique	PRU	TE	Hypertension	Diabetes mellitus	Dyslipidemia	Smoking	Ischemic stroke history	procedure duration(min)
51	Female	ICA	4.5	3.6	Simple	355	0	1	0	0	0	0	110
62	Male	Posterior	10.1	6.8	Stent-assisted	247	1	1	0	0	0	1	111
72	Female	ICA	7.0	4.1	Stent-assisted	50	0	1	0	0	0	0	104
49	Female	Posterior	14.3	14.3	Stent-assisted	40	0	0	0	0	0	0	156
57	Female	ICA	8.6	7.7	Stent-assisted	353	0	0	1	1	0	0	100
73	Female	ICA	11.0	3.7	Stent-assisted	222	0	1	0	0	0	0	130
47	Male	ICA	7.6	4.2	Simple	284	0	0	0	0	0	0	115
64	Female	ICA	5.5	2.9	Stent-assisted	383	0	1	0	1	0	0	110
59	Female	ICA	7.0	3.9	Stent-assisted	97	0	0	0	0	0	0	95
65	Female	ICA	5.9	4.0	Simple	281	0	0	0	0	0	0	135
72	Female	ACA/MCA	3.9	3.0	Simple	294	0	1	0	0	0	0	112
60	Male	ICA	5.3	3.0	Simple	234	0	1	0	1	0	0	80
72	Female	ICA	10.8	6.0	Stent-assisted	291	1	1	0	1	0	0	235
64	Female	ICA	4.4	1.6	Balloon-assisted	133	0	1	0	0	0	0	108
70	Female	Posterior	13.6	6.3	Stent-assisted	179	0	1	1	1	0	0	157
57	Female	Posterior	10.8	4.7	Stent-assisted	62	0	1	0	1	0	0	225
69	Female	ICA	4.9	3.3	Stent-assisted	281	0	1	0	0	0	0	131
66	Female	Posterior	5.4	4.9	Stent-assisted	372	0	1	0	0	0	0	95
65	Female	ICA	4.9	2.8	Balloon-assisted	327	1	1	1	0	1	0	243
57	Female	ICA	10.7	6.1	Stent-assisted	5	0	1	0	1	0	0	158
58	Female	ICA	3.7	3.6	Balloon-assisted	75	0	0	0	1	0	0	83
67	Male	ACA/MCA	4.7	3.7	Simple	292	0	1	0	0	0	0	109
50	Female	ICA	7.0	4.3	Stent-assisted	7	0	0	0	0	0	0	129
65	Female	ACA/MCA	5.2	4.0	Stent-assisted	380	1	1	0	0	0	0	125
70	Male	ACA/MCA	6.3	2.9	Simple	372	0	1	0	0	0	0	95
79	Female	Posterior	5.5	4.4	Stent-assisted	313	0	1	0	0	0	0	125
68	Female	ICA	3.1	2.2	Simple	149	0	1	0	0	1	0	223
69	Female	ICA	5.5	2.0	Balloon-assisted	225	0	1	0	0	0	0	107
73	Female	ICA	4.1	4.0	Simple	295	0	1	1	0	0	0	83
55	Female	Posterior	10.8	7.6	Stent-assisted	141	0	1	0	0	0	0	240
65	Male	ICA	7.2	4.3	Simple	10	0	1	0	0	0	0	155
62	Female	ICA	5.7	4.3	Stent-assisted	308	0	0	0	0	1	0	216
59	Male	ICA	4.7	3.3	Simple	224	0	1	0	0	0	0	125
64	Female	ACA/MCA	3.3	2.4	Simple	331	0	0	0	0	1	0	113
54	Female	ICA	21.5	7.0	Stent-assisted	211	0	0	0	0	0	0	233
88	Female	ICA	26.7	4.7	Stent-assisted	343	0	1	0	0	0	0	306
48	Male	Posterior	7.2	5.2	Balloon-assisted	286	0	1	1	0	1	0	200
68	Female	Posterior	7.5	4.5	Stent-assisted	328	0	0	0	0	0	0	117
57	Male	Posterior	5.0	2.1	Stent-assisted	175	0	1	0	1	1	1	224
55	Female	Posterior	6.0	4.1	Stent-assisted	207	0	1	0	0	0	0	119
45	Female	ICA	5.9	5.1	Stent-assisted	166	0	0	0	0	0	0	204
62	Male	ACA/MCA	3.4	2.4	Simple	258	0	0	1	1	0	0	159
60	Female	ACA/MCA	5.5	3.5	Stent-assisted	258	0	1	0	1	0	1	158
78	Male	Posterior	12.4	12.4	Stent-assisted	387	1	1	0	0	1	0	226
74	Male	ACA/MCA	5.7	4.3	Simple	232	0	1	0	0	1	0	184
64	Male	ACA/MCA	7.0	4.1	Stent-assisted	69	0	1	0	0	0	0	203
55	Female	ICA	10.2	5.1	Stent-assisted	170	0	0	0	0	0	0	192
37	Male	Posterior	3.2	2.8	Balloon-assisted	186	0	1	0	1	0	0	87
60	Female	Posterior	9.8	8.5	Stent-assisted	131	0	1	0	0	1	0	265
64	Female	ACA/MCA	3.7	3.5	Simple	288	0	0	0	0	0	0	98
74	Female	ICA	4.4	2.3	Balloon-assisted	233	0	1	0	0	0	1	80
64	Female	ICA	4.3	4.1	Simple	263	0	0	0	0	0	0	110
60	Female	ICA	4.2	1.5	Stent-assisted	224	0	0	0	0	0	0	127
56	Male	Posterior	7.1	7.1	Stent-assisted	173	0	1	0	0	1	0	148
76	Female	ACA/MCA	5.9	2.8	Simple	243	0	1	0	0	1	0	110
75	Female	ICA	7.0	6.0	Stent-assisted	417	0	1	0	1	0	0	96
73	Female	ICA	6.9	3.8	Balloon-assisted	164	0	0	0	0	0	0	97
70	Male	ICA	5.7	3.7	Stent-assisted	331	0	1	0	1	1	1	148
36	Female	ICA	18.4	18.4	Stent-assisted	142	0	0	0	0	0	0	140
53	Female	ICA	3.8	3.0	Balloon-assisted	236	0	0	0	0	0	0	76
61	Female	ACA/MCA	5.1	2.4	Balloon-assisted	151	0	0	0	1	0	0	110
58	Male	ICA	5.2	2.1	Balloon-assisted	233	0	1	0	0	0	0	73
39	Female	ICA	4.1	3.1	Balloon-assisted	81	0	1	0	0	0	0	117
41	Female	Posterior	12.3	12.3	Stent-assisted	291	0	1	0	0	0	0	125
47	Female	ICA	7.1	5.1	Stent-assisted	188	0	1	0	0	0	1	183
75	Female	ACA/MCA	3.7	2.3	Simple	342	0	0	0	0	0	0	109
56	Male	Posterior	11.6	7.7	Stent-assisted	144	0	0	0	0	1	0	184
53	Female	ICA	5.1	2.0	Balloon-assisted	212	0	1	0	0	0	0	155
76	Female	ACA/MCA	5.6	1.9	Simple	243	1	1	0	0	1	0	123
72	Female	ICA	7.2	4.6	Balloon-assisted	330	0	0	0	0	0	0	115
62	Female	ICA	7.4	4.9	Stent-assisted	253	0	0	0	0	0	0	125
38	Female	ICA	5.1	3.8	Balloon-assisted	339	0	0	0	0	0	0	83
61	Female	ICA	4.2	2.6	Simple	315	0	0	0	0	0	0	82
46	Female	ICA	4.6	3.0	Balloon-assisted	243	0	0	0	0	0	0	93
50	Male	ICA	4.1	2.9	Balloon-assisted	154	0	1	0	0	0	0	65
65	Female	Posterior	3.3	3.1	Simple	327	1	1	0	0	0	0	243
64	Female	ICA	7.3	2.8	Balloon-assisted	368	0	1	0	1	0	0	85
76	Male	ICA	4.6	4.5	Stent-assisted	139	0	1	0	0	0	0	100
51	Female	ICA	4.0	2.1	Simple	237	0	1	0	0	0	0	88
63	Male	ICA	6.2	5.1	Stent-assisted	291	0	1	0	1	1	0	100
64	Female	ICA	6.8	6.8	Stent-assisted	122	0	1	0	0	0	0	157
36	Female	ICA	5.7	3.7	Stent-assisted	97	1	0	0	0	0	0	117
43	Male	ACA/MCA	4.0	2.4	Simple	236	0	1	0	0	0	0	74
25	Female	ICA	5.2	4.3	Balloon-assisted	175	0	0	0	0	0	0	125
62	Female	ICA	8.2	4.8	Stent-assisted	235	0	0	0	0	0	0	137
23	Female	ICA	3.7	2.5	Simple	158	0	0	0	0	1	0	127
60	Male	ICA	4.4	2.9	Simple	97	0	0	0	0	1	0	98
76	Female	Posterior	6.5	3.1	Balloon-assisted	231	0	1	0	1	0	0	135
76	Female	ACA/MCA	3.6	2.3	Simple	250	0	1	0	0	0	0	128
71	Female	ICA	10.5	5.3	Balloon-assisted	279	0	1	0	0	0	0	245
77	Female	ICA	3.4	2.7	Simple	95	0	1	1	1	0	0	145
53	Female	ICA	5.4	2.5	Simple	68	0	0	0	0	1	0	140
44	Female	ICA	6.9	2.6	Simple	226	0	0	0	0	0	0	130
73	Female	ICA	8.5	5.5	Stent-assisted	247	0	1	1	1	0	0	135
70	Female	ACA/MCA	10.3	4.4	Stent-assisted	290	0	1	0	0	0	0	275
51	Female	ICA	5.9	3.5	Balloon-assisted	313	0	0	0	0	0	0	183
55	Male	ICA	10.0	5.0	Balloon-assisted	142	0	0	0	0	1	0	175
72	Male	Posterior	15.0	6.8	Stent-assisted	185	0	1	0	1	1	0	377
59	Male	ACA/MCA	4.8	3.7	Simple	250	0	1	0	1	0	1	160
67	Male	ACA/MCA	3.3	2.6	Simple	217	0	1	0	1	1	1	90
58	Female	Posterior	5.2	4.5	Stent-assisted	197	0	1	0	0	0	0	174
77	Female	ICA	9.1	4.7	Balloon-assisted	203	0	0	0	0	0	0	80
77	Female	ICA	5.9	2.4	Balloon-assisted	225	0	1	0	1	0	0	202
52	Female	Posterior	8.2	8.2	Stent-assisted	150	0	0	0	0	0	0	105
60	Female	ICA	5.0	3.4	Stent-assisted	301	0	0	0	0	0	0	133

61	Female	ICA	5.1	3.2	Simple	222	0	1	0	0	0	0	120
72	Female	ICA	4.8	2.6	Simple	276	0	1	0	0	0	0	110
63	Female	ICA	5.1	3.2	Simple	275	0	1	1	1	0	0	91
58	Female	Posterior	7.5	7.5	Stent-assisted	307	1	1	0	1	0	0	145
78	Female	ICA	5.1	2.9	Simple	382	0	1	0	0	0	0	100
74	Female	ICA	5.4	4.0	Balloon-assisted	229	0	0	0	0	0	0	126
83	Male	Posterior	11.8	6.3	Stent-assisted	181	0	1	0	0	0	0	158
58	Male	Posterior	3.4	2.9	Balloon-assisted	202	0	1	0	0	1	0	120
54	Female	ICA	7.1	5.2	Stent-assisted	346	0	0	0	1	0	0	194
74	Female	ICA	7.5	4.5	Stent-assisted	388	1	0	0	1	0	0	152
57	Male	ACA/MCA	4.4	2.3	Stent-assisted	175	0	1	0	1	1	1	224
39	Female	ICA	4.7	3.3	Balloon-assisted	378	0	1	0	0	0	0	134
75	Male	ICA	10.8	5.4	Stent-assisted	353	0	0	0	1	0	0	113
60	Male	Posterior	3.8	3.6	Stent-assisted	169	0	1	1	1	1	0	164
76	Female	ICA	7.2	5.1	Stent-assisted	238	0	1	0	0	0	0	130
57	Female	ICA	6.5	3.4	Balloon-assisted	210	0	0	0	1	0	0	85
59	Female	Posterior	3.6	3.2	Balloon-assisted	232	0	1	0	1	1	0	100
44	Male	ICA	5.9	4.4	Stent-assisted	313	0	0	0	1	1	0	195
46	Male	ICA	3.4	3.0	Balloon-assisted	215	0	0	0	1	1	0	85
43	Female	ICA	5.7	3.2	Balloon-assisted	293	0	0	0	0	1	0	120
68	Male	ACA/MCA	6.5	4.0	Simple	237	0	1	0	1	0	0	183
48	Female	ICA	5.5	3.6	Balloon-assisted	219	0	0	0	0	0	0	124
77	Female	ICA	15.0	5.3	Stent-assisted	218	1	1	0	1	1	0	267
69	Female	Posterior	5.5	5.2	Stent-assisted	256	0	0	0	1	0	0	122
72	Female	ICA	7.0	4.4	Balloon-assisted	175	0	1	0	0	0	0	155
72	Female	ICA	25.0	5.5	Stent-assisted	368	1	1	0	1	0	0	264
74	Male	ICA	3.4	2.7	Balloon-assisted	237	0	1	0	0	0	0	100
45	Female	Posterior	4.2	3.0	Balloon-assisted	283	0	0	0	0	0	0	110
78	Female	Posterior	6.3	5.0	Stent-assisted	433	1	1	0	0	0	0	112
55	Female	ICA	18.0	4.0	Balloon-assisted	220	0	1	0	1	0	0	200
75	Female	ICA	7.9	4.0	Balloon-assisted	299	0	1	1	1	0	0	180
58	Male	ACA/MCA	8.3	2.2	Balloon-assisted	169	0	1	0	1	1	0	112
47	Female	ICA	4.1	2.2	Balloon-assisted	249	0	0	0	0	0	0	102
82	Female	ICA	6.3	4.1	Stent-assisted	55	0	1	0	1	0	0	127
62	Female	ICA	5.4	4.6	Stent-assisted	134	0	0	0	1	0	0	100
65	Female	Posterior	3.3	3.1	Simple	327	1	1	1	0	1	0	243
56	Female	ICA	25.0	9.5	Stent-assisted	287	1	0	0	0	0	0	332
65	Female	ACA/MCA	5.6	3.3	Balloon-assisted	165	0	0	0	1	0	0	175
63	Female	ICA	6.3	3.2	Simple	240	0	0	0	0	0	0	121
53	Female	Posterior	4.9	3.6	Stent-assisted	425	0	1	0	0	1	0	105
66	Female	ICA	6.5	5.2	Stent-assisted	275	0	0	0	1	0	0	145
66	Male	ICA	5.1	3.4	Balloon-assisted	158	0	1	0	1	1	0	120
71	Female	ICA	10.7	8.4	Stent-assisted	128	0	1	0	1	0	0	198
69	Female	ICA	4.4	2.2	Balloon-assisted	80	0	1	0	1	0	0	171
56	Female	ICA	5.4	4.5	Balloon-assisted	220	0	0	0	0	1	0	121
76	Female	ICA	5.1	3.5	Balloon-assisted	112	1	1	0	0	0	0	157
58	Female	ICA	3.6	2.7	Balloon-assisted	212	0	1	0	1	0	0	110
74	Female	ACA/MCA	9.0	4.2	Balloon-assisted	292	0	0	0	0	1	0	142
59	Female	ACA/MCA	6.3	3.9	Simple	334	0	0	0	0	1	0	209
48	Male	Posterior	5.6	1.6	Balloon-assisted	139	0	1	0	0	1	0	180
74	Female	ACA/MCA	5.7	3.2	Stent-assisted	292	0	0	0	0	1	0	185
77	Female	ICA	4.5	4.4	Balloon-assisted	165	0	0	0	1	1	0	180
69	Female	ICA	6.4	3.4	Balloon-assisted	225	0	1	0	1	0	0	140
80	Male	ICA	15.5	5.9	Stent-assisted	118	0	0	0	0	1	0	246
26	Female	ICA	2.9	1.6	Balloon-assisted	244	0	0	0	0	1	0	125
57	Female	ICA	5.3	3.0	Balloon-assisted	230	0	0	0	0	0	0	90
66	Female	ACA/MCA	7.0	3.5	Stent-assisted	253	0	1	0	1	0	0	184
57	Female	ICA	3.6	2.2	Simple	254	0	1	0	1	0	0	94
59	Female	ACA/MCA	7.4	3.1	Stent-assisted	273	1	1	1	1	1	0	120
59	Male	Posterior	8.8	4.5	Stent-assisted	165	0	1	0	0	1	0	159
73	Female	ICA	12.9	6.3	Stent-assisted	351	0	1	0	0	0	0	190
72	Female	ICA	5.4	4.7	Balloon-assisted	340	1	0	0	0	0	0	140
53	Female	ICA	7.0	6.3	Stent-assisted	138	0	0	0	0	0	0	109
60	Female	ICA	7.5	3.5	Balloon-assisted	272	0	1	0	1	1	0	187
65	Female	ICA	6.0	3.6	Balloon-assisted	339	0	0	0	0	0	0	95
50	Female	ACA/MCA	4.8	4.3	Stent-assisted	167	0	1	0	1	0	0	191
66	Female	ACA/MCA	8.0	6.9	Balloon-assisted	185	0	1	0	1	0	0	190
76	Female	ICA	4.1	4.1	Stent-assisted	278	0	0	0	1	0	0	174
72	Female	ICA	10.2	5.7	Simple	263	0	1	1	1	0	0	142
64	Female	ICA	8.0	5.6	Stent-assisted	268	0	0	0	1	0	0	270
63	Female	ICA	7.8	4.8	Stent-assisted	296	0	1	0	0	0	0	175
76	Female	ACA/MCA	5.6	1.9	Simple	243	1	1	0	0	1	0	123
50	Female	Posterior	8.4	4.6	Balloon-assisted	291	0	0	0	0	0	0	180
70	Female	ICA	5.7	3.5	Balloon-assisted	248	0	0	0	1	0	0	100
39	Female	ICA	8.8	6.0	Stent-assisted	139	0	0	0	0	0	0	190
54	Female	ICA	22.2	5.1	Stent-assisted	306	0	1	0	0	0	0	310
42	Female	ICA	6.5	3.5	Stent-assisted	286	0	1	0	0	0	0	210
49	Male	ACA/MCA	4.3	1.9	Balloon-assisted	219	0	1	0	1	1	0	195
71	Female	ICA	7.5	5.0	Stent-assisted	317	0	1	0	0	0	0	125
79	Male	ACA/MCA	7.5	4.5	Balloon-assisted	173	0	1	0	1	1	0	85
66	Female	ICA	7.0	4.4	Stent-assisted	273	0	0	0	1	0	0	151
35	Female	ICA	6.5	3.6	Stent-assisted	370	0	0	0	1	0	0	165
41	Female	ICA	3.8	3.3	Stent-assisted	156	0	0	0	0	1	0	340
55	Female	ICA	6.5	2.6	Balloon-assisted	331	0	0	0	1	1	0	115
77	Female	ICA	7.1	3.8	Simple	193	0	0	0	0	1	0	145
75	Female	Posterior	4.4	4.2	Balloon-assisted	248	0	1	0	1	0	0	212
76	Female	Posterior	8.2	6.5	Stent-assisted	248	0	1	0	0	0	0	135
36	Female	ACA/MCA	5.2	3.5	Simple	180	0	0	0	0	1	0	217
58	Female	ACA/MCA	5.5	3.9	Balloon-assisted	242	0	1	1	1	0	0	177
76	Female	ICA	3.1	2.5	Simple	198	0	0	0	0	0	0	135
77	Male	ICA	5.0	3.0	Balloon-assisted	116	0	1	0	0	1	0	80
56	Female	ICA	7.4	4.8	Balloon-assisted	274	0	0	0	1	1	0	188
65	Female	ACA/MCA	3.5	3.0	Balloon-assisted	298	0	1	0	0	1	1	190
74	Male	ICA	9.5	4.9	Balloon-assisted	205	0	1	0	0	1	0	222
51	Male	Posterior	10.6	8.0	Stent-assisted	115	0	1	0	0	0	0	302
38	Female	ICA	3.8	3.0	Balloon-assisted	265	0	0	0	1	1	0	195
69	Female	ICA	4.0	3.0	Balloon-assisted	343	0	0	0	0	0	0	211
47	Male	ACA/MCA	6.0	5.4	Simple	160	1	1	1	1	1	0	150
72	Male	Posterior	14.9	6.8	Stent-assisted	185	0	1	0	1	1	0	377
68	Female	ACA/MCA	4.8	3.7	Simple	149	0	1	0	0	1	0	223
77	Female	ACA/MCA	6.5	4.0	Stent-assisted	162	0	1	0	1	0	0	310
34	Male	ICA	4.3	3.1	Balloon-assisted	194	0	1	0	1	1	0	243
34	Male	ICA	3.8	3.4	Balloon-assisted	194	0	1	0	1	1	0	243
60	Female	Posterior	2.6	2.3	Balloon-assisted	156	1	1	0	1	0	0	120
70	Female	ICA	7.7	3.6	Balloon-assisted	328	1	1	0	0	0	0	231
73	Female	ICA	13.7	5.3	Stent-assisted	227	0	1	0	0	0	0	150
76	Female	ACA/MCA	4.7	3.0	Balloon-assisted	301	0	0	0	0	0	0	140

78	Female	Posterior	5.9	4.0	Simple	121	0	0	0	0	0	0	142
45	Female	ICA	5.4	3.3	Stent-assisted	169	0	1	0	0	1	0	130
66	Female	ICA	7.0	2.3	Balloon-assisted	192	0	0	0	0	0	0	143
55	Female	ICA	4.5	2.4	Balloon-assisted	200	0	1	0	1	1	0	98
64	Female	ICA	6.3	5.1	Stent-assisted	155	0	0	0	0	1	0	195
73	Female	ICA	6.5	3.5	Balloon-assisted	294	0	1	0	1	0	0	207
43	Female	Posterior	6.2	2.7	Simple	186	0	0	0	0	0	0	192
26	Female	ICA	3.6	3.5	Stent-assisted	5	0	0	0	0	0	0	120
60	Female	ICA	5.3	3.6	Simple	229	0	1	1	1	0	0	113
61	Female	ICA	6.0	3.0	Balloon-assisted	226	0	0	0	0	0	0	80
75	Male	ICA	5.5	3.6	Balloon-assisted	242	0	0	1	0	1	0	131
41	Female	ICA	4.8	3.2	Stent-assisted	156	0	0	0	0	1	0	340
73	Male	ACA/MCA	8.9	3.4	Simple	259	0	1	0	0	1	0	190
57	Male	Posterior	6.0	3.6	Simple	76	0	1	1	1	1	0	161
41	Female	ICA	6.2	4.0	Simple	105	0	0	0	0	0	0	173
56	Female	ACA/MCA	4.5	2.6	Simple	47	0	1	0	0	1	0	128
58	Male	ACA/MCA	6.0	5.9	Stent-assisted	64	0	0	0	1	1	0	311
64	Female	ACA/MCA	5.3	3.0	Balloon-assisted	219	0	0	0	1	0	0	176
39	Female	ICA	5.2	4.3	Balloon-assisted	183	0	1	0	0	0	0	145
74	Female	ICA	4.6	3.1	Balloon-assisted	252	0	1	0	1	0	0	80
68	Female	ACA/MCA	4.0	2.8	Balloon-assisted	267	0	1	0	1	0	0	172
65	Female	ACA/MCA	4.8	2.5	Simple	213	0	1	0	0	0	0	123
58	Female	ICA	5.2	3.2	Simple	180	0	0	0	0	0	0	155
71	Female	ICA	5.4	2.8	Balloon-assisted	147	0	1	0	1	0	0	95
73	Female	ICA	9.6	4.6	Stent-assisted	163	0	1	0	1	0	0	107
66	Female	ICA	5.2	4.0	Stent-assisted	323	0	0	0	0	0	0	70
64	Female	Posterior	14.0	7.9	Stent-assisted	229	0	1	0	0	0	0	86
66	Female	ICA	7.6	4.4	Balloon-assisted	245	0	1	0	0	0	0	72
75	Female	ACA/MCA	5.7	3.9	Stent-assisted	144	0	0	0	0	0	0	157
55	Female	Posterior	9.9	7.4	Stent-assisted	390	0	1	1	1	0	0	125
72	Female	ACA/MCA	6.5	3.3	Simple	276	0	1	0	0	0	0	110
36	Female	ICA	4.2	2.1	Balloon-assisted	235	0	0	0	0	0	0	52
71	Male	Posterior	18.0	10.3	Stent-assisted	188	0	0	0	0	1	0	165
50	Female	ICA	5.1	2.4	Balloon-assisted	253	0	1	0	1	1	0	85
55	Female	ACA/MCA	7.2	4.8	Stent-assisted	296	0	1	1	1	0	0	210
50	Male	ICA	4.6	2.8	Balloon-assisted	152	0	0	0	0	1	0	100
74	Female	ICA	9.1	5.7	Stent-assisted	182	0	0	0	0	0	0	75
48	Female	ICA	5.0	3.3	Balloon-assisted	120	0	1	0	0	0	0	40
74	Female	ICA	8.8	4.8	Stent-assisted	127	0	1	0	0	0	0	120
73	Female	ACA/MCA	11.2	5.3	Stent-assisted	223	0	1	0	1	0	0	120
57	Male	ICA	9.1	5.8	Stent-assisted	142	0	1	0	0	1	0	92
64	Female	Posterior	13.2	5.4	Simple	78	0	1	0	0	0	0	102
33	Female	ICA	6.1	3.7	Balloon-assisted	333	0	0	0	0	1	0	105
74	Female	ICA	6.4	3.3	Balloon-assisted	239	0	0	0	1	1	0	90
42	Female	ICA	5.6	3.0	Simple	196	0	0	0	1	0	0	100
65	Female	ICA	4.9	2.8	Balloon-assisted	327	1	1	0	0	0	0	243
55	Male	Posterior	7.6	7.6	Stent-assisted	264	0	1	0	0	1	0	91
67	Female	ACA/MCA	5.6	2.4	Stent-assisted	139	0	1	0	0	0	0	134
44	Female	ICA	8.3	4.2	Stent-assisted	8	0	0	0	0	0	0	87
75	Female	ICA	7.0	4.7	Stent-assisted	203	0	0	0	0	0	0	95
46	Male	ICA	4.5	3.0	Stent-assisted	265	0	1	1	0	1	0	150
52	Female	ICA	4.0	3.0	Simple	182	0	1	0	0	1	0	150
52	Female	ICA	4.4	3.6	Balloon-assisted	182	0	1	0	0	1	0	150
66	Male	ACA/MCA	5.1	2.4	Simple	236	0	1	0	0	1	0	97
52	Female	ICA	10.8	4.5	Stent-assisted	102	0	0	0	0	1	0	164
38	Female	ICA	5.0	2.9	Balloon-assisted	264	0	0	0	0	0	0	90
75	Female	ICA	7.3	3.9	Stent-assisted	139	0	0	0	1	0	0	180
68	Female	ICA	7.1	6.9	Balloon-assisted	149	0	1	0	0	1	0	223
54	Male	Posterior	24.0	24.0	Stent-assisted	6	1	1	1	1	1	1	285
46	Female	ICA	4.9	2.5	Simple	331	0	0	0	0	1	0	72
79	Female	ICA	8.3	5.2	Stent-assisted	66	0	0	0	1	0	0	188
75	Male	ACA/MCA	9.1	4.4	Stent-assisted	216	1	1	0	1	1	0	205
39	Female	Posterior	14.4	7.3	Stent-assisted	284	1	0	0	0	0	0	400
69	Female	ICA	7.2	4.4	Stent-assisted	257	0	0	0	0	0	0	150
41	Female	ICA	2.8	2.3	Stent-assisted	156	0	0	0	0	1	0	340
72	Female	ICA	11.2	5.4	Stent-assisted	148	0	1	0	1	0	0	75
57	Female	ICA	5.8	2.5	Simple	187	0	0	0	0	0	0	57
56	Female	ICA	9.0	3.2	Balloon-assisted	84	0	0	0	0	0	0	95
46	Male	ICA	6.0	5.3	Stent-assisted	231	0	0	0	0	0	0	150
58	Male	ACA/MCA	5.3	4.2	Stent-assisted	106	0	0	0	0	1	0	170
69	Male	ACA/MCA	6.7	5.9	Stent-assisted	69	0	1	0	1	0	0	165
60	Female	Posterior	5.7	4.5	Stent-assisted	284	0	0	0	0	0	0	74
76	Female	Posterior	4.5	2.9	Stent-assisted	308	0	1	1	0	0	0	130
44	Female	ICA	5.5	4.0	Stent-assisted	55	0	0	0	0	0	0	96
78	Female	ICA	11.0	9.0	Stent-assisted	247	0	1	0	0	0	0	146
63	Female	ICA	4.4	3.7	Stent-assisted	221	0	1	1	1	0	0	141
66	Female	ICA	7.9	5.1	Stent-assisted	170	0	1	0	1	0	1	103
66	Female	ACA/MCA	10.0	5.6	Stent-assisted	234	1	1	0	0	1	0	139
42	Female	ICA	6.0	4.5	Stent-assisted	169	0	0	0	0	0	0	123
59	Female	ACA/MCA	5.0	2.2	Simple	183	0	1	0	0	1	0	120
64	Female	ICA	7.3	6.4	Stent-assisted	230	0	1	0	0	0	0	240
75	Female	ICA	7.4	2.8	Simple	215	0	1	1	0	0	0	150
70	Female	ICA	6.5	4.5	Balloon-assisted	144	0	1	1	1	1	0	90
61	Female	ICA	5.6	3.1	Simple	210	0	1	0	0	1	0	82
67	Female	ACA/MCA	3.8	3.2	Stent-assisted	171	0	1	0	0	0	0	140
78	Female	ICA	9.3	4.9	Balloon-assisted	79	0	1	0	0	1	0	180
73	Female	ACA/MCA	5.5	2.8	Simple	269	0	1	0	0	0	1	133
57	Female	ICA	5.3	4.1	Stent-assisted	238	0	0	0	0	1	0	145
61	Female	ICA	4.4	2.8	Simple	210	0	1	0	0	1	0	110
85	Female	ICA	13.9	3.2	Simple	237	0	1	0	0	0	0	123
69	Female	Posterior	6.1	3.9	Stent-assisted	191	0	1	0	0	0	0	171
67	Male	Posterior	14.1	7.2	Stent-assisted	50	0	1	1	1	1	0	225
58	Male	Posterior	7.8	6.2	Stent-assisted	254	0	0	0	0	1	0	86
68	Female	ICA	11.2	4.4	Stent-assisted	257	0	0	0	1	1	0	265
77	Female	ICA	5.5	4.5	Balloon-assisted	165	0	0	0	1	1	0	180
67	Female	Posterior	6.2	3.6	Stent-assisted	236	0	1	0	0	0	0	88
76	Female	ACA/MCA	8.0	4.5	Stent-assisted	274	0	1	0	0	0	0	160
54	Female	ICA	5.4	3.6	Balloon-assisted	177	0	0	0	1	1	0	100
79	Female	Posterior	8.0	5.4	Stent-assisted	12	0	1	0	0	0	0	185
66	Female	ICA	5.1	2.2	Simple	289	0	1	0	0	0	0	105
51	Female	ICA	7.4	4.1	Stent-assisted	155	0	1	0	1	0	0	134
59	Female	ACA/MCA	6.0	4.6	Stent-assisted	223	0	0	0	0	0	0	150
75	Female	ICA	5.9	3.0	Balloon-assisted	299	0	1	1	1	1	0	103
75	Male	Posterior	7.0	5.6	Stent-assisted	164	0	1	0	1	1	1	120
65	Female	Posterior	5.1	3.8	Stent-assisted	136	0	0	0	0	0	0	60
60	Female	ICA	5.9	4.1	Stent-assisted	163	0	1	0	1	0	0	150
72	Female	ACA/MCA	4.6	2.7	Simple	247	0	1	0	0	0	0	115

81	Female	ACA/MCA	5.6	3.0	Simple	249	0	1	0	0	0	0	157
57	Female	ICA	7.1	4.0	Stent-assisted	202	0	0	0	0	0	0	81
65	Female	ICA	6.5	3.4	Stent-assisted	246	0	1	0	0	0	0	115
52	Male	Posterior	12.6	12.6	Stent-assisted	114	0	1	0	0	0	0	151
76	Female	ICA	6.1	2.9	Simple	172	0	1	0	1	1	0	72
43	Male	ACA/MCA	6.0	4.7	Stent-assisted	34	0	0	1	1	1	0	161
45	Female	ICA	6.8	4.7	Stent-assisted	102	0	0	0	0	0	0	100
70	Female	ICA	7.0	4.3	Stent-assisted	139	0	0	0	0	0	0	131
82	Female	ACA/MCA	6.5	3.2	Stent-assisted	263	0	1	0	1	0	0	201
69	Female	ICA	6.0	4.3	Stent-assisted	218	0	0	1	0	0	0	65
75	Female	ICA	6.0	2.7	Stent-assisted	249	1	1	1	0	0	0	119
41	Female	ACA/MCA	4.3	2.7	Simple	156	0	0	0	0	1	0	340
46	Female	Posterior	4.0	3.0	Stent-assisted	233	0	0	0	0	0	0	105
77	Male	ICA	11.7	4.2	Stent-assisted	204	0	1	1	0	1	1	90
49	Female	ACA/MCA	4.8	2.7	Simple	155	0	1	0	0	0	0	113
58	Female	ICA	4.8	2.7	Simple	112	0	1	0	0	1	0	92
51	Male	ACA/MCA	5.5	3.3	Simple	197	0	1	1	1	1	0	92
73	Male	ACA/MCA	5.1	3.3	Simple	179	0	0	1	0	1	0	136
74	Female	ICA	8.0	7.5	Stent-assisted	171	0	1	0	1	0	0	117
43	Female	ICA	6.9	4.1	Stent-assisted	212	0	0	0	0	0	0	171
41	Male	Posterior	7.5	4.2	Stent-assisted	150	0	0	0	0	1	0	145
55	Female	ICA	4.4	2.9	Simple	132	0	0	0	1	0	0	132
66	Female	ICA	5.8	4.2	Stent-assisted	234	0	1	0	0	1	0	135
77	Male	ACA/MCA	9.5	6.3	Stent-assisted	196	0	1	0	0	1	0	189
54	Female	ICA	5.1	3.3	Stent-assisted	207	0	0	0	0	0	0	118
67	Female	ICA	5.0	2.6	Simple	294	0	0	0	1	1	0	78
